# Peripheral Interleukin-18 is negatively correlated with abnormal brain activity in patients with depression: a resting-state fMRI study

**DOI:** 10.1186/s12888-022-04176-8

**Published:** 2022-08-05

**Authors:** Xiangdong Du, Siyun Zou, Yan Yue, Xiaojia Fang, Yuxuan Wu, Siqi Wu, Haitao Wang, Zhe Li, Xueli Zhao, Ming Yin, Gang Ye, Hongyan Sun, Xiaochu Gu, Xiaobin Zhang, Zhigang Miao, Jeff Wang Jin, Hanjing Emily Wu, Yansong Liu, Xingshun Xu

**Affiliations:** 1 Department of Neurology, the Second Affiliated Hospital of Soochow University, Suzhou, 215004 China; 2 Suzhou Guangji Hospital, The Affiliated Guangji Hospital of Soochow University, Suzhou, 215004 China; 3 Medical College of Soochow University, Suzhou, China; 4 Xuzhou Medical University, Xuzhou, China; 5 School of Psychology and Mental Health, North China University of Science and Technology, Tangshan, China; 6 Department of Psychiatry and Behavioral Sciences, The University of Texas Health Science Center at Houston, Houston, TX USA

**Keywords:** Depression, Interleukin-18, Degree centrality, Correlation

## Abstract

**Background:**

Interleukin-18 (IL-18) may participate in the development of major depressive disorder, but the specific mechanism remains unclear. This study aimed to explore whether IL-18 correlates with areas of the brain associated with depression.

**Methods:**

Using a case–control design, 68 subjects (34 patients and 34 healthy controls) underwent clinical assessment, blood sampling, and resting-state functional Magnetic Resonance Imaging (fMRI). The total Hamilton depression-17 (HAMD-17) score was used to assess depression severity. Enzyme-linked immunosorbent assay (ELISA) was used to detect IL-18 levels. Rest-state fMRI was conducted to explore spontaneous brain activity.

**Results:**

The level of IL-18 was higher in patients with depression in comparison with healthy controls. IL-18 was negatively correlated with degree centrality of the left posterior cingulate gyrus in the depression patient group, but no correlation was found in the healthy control group.

**Conclusion:**

This study suggests the involvement of IL-18 in the pathophysiological mechanism for depression and interference with brain activity.

## Introduction

Depression is a very common disease with a global prevalence of 4% [1] and lifetime prevalence in China of 6.8% [2]. Although the exact mechanism of depression remains unknown, the classic monoamine, glutamate, hypothalamic–pituitary–adrenal axis, and neurotrophic hypotheses propose several pathophysiological etiologies. However, these theories have not fully revealed the pathogenesis of depression. Several studies suggest the important role of inflammatory immune factors in depression development [3–6].

Interleukin-18 (IL-18) is an inflammatory factor involved in immune function regulation. Prior studies suggest the involvement of IL-18 in depression-like behaviors in mice [7]. Additionally, several clinical studies report increased IL-18 peripheral blood levels in patients with depression in comparison to healthy individuals, and Al-Hakeim.et al. found non-steroidal anti-inflammatory drug ibuprofen can decrease the levels of IL—18 of patients with depression, when it is used of an auxiliary treatment of depression [8–11]. Furthermore, our prior study demonstrated the involvement of IL-18 in the mechanism for post-stroke depression (Wu et al., 2020). However, the role of IL-18 remains unclear in depressive behaviors and warrants further investigation. Although animal studies have shown that abnormal IL-18 level may lead to changes in the structure of the hippocampus and the appearance of “depression” in mice [7], few studies have explored the relationship between IL-18 and brain functional activity in patients with depression.

Resting-state brain imaging can be used to indirectly analyze spontaneous brain activity changes during non-task activities through detection of the blood oxygen concentration – a method used widely in exploring depression etiologies. Degree centrality (DC), an important resting state brain imaging indicator, is one of the graph analysis methods. It is the number or weighted sum of a certain cortical vertex and the whole cortical vertex functional connectivity (FC) above a specific threshold, which is closely related to whole-brain functional connectivity and can reflect the importance of specific brain node or area relative to the whole brain network [12, 13]. In recent years, DC has been used to study characteristics of brain imaging in depression. Researches show that the DC values in the prefrontal cortex of the default network and the left caudate nucleus are statistically significantly associated with the severity of depression in patients with depression, in other words, DC is abnormal in patients with depression [14–17]. However, there is no study to explore the relationship between these two indicators that are closely related to the occurrence and development of depression.

In the current study, we measured serum IL-18 levels and analyzed its relationship with depression-related brain region activity in patients and healthy controls. To our knowledge, this is the first study to explore the correlation between IL-18 and DC.

## Methods

### Patients and controls

A case–control study was conducted from June 2019 to November 2020 in the Guangji Hospital affiliated with Soochow University (Suzhou City, China). Recruited patients met the following inclusion criteria: (1) inpatient adults (20 to 65 years old) and (2) diagnosed with major depressive disorder by Structured Clinical Interview for DSM-5 (SCID-5). Subjects were excluded if they were: (1) unable to undergo MRI scanning, (2) left-handed, (3) pregnant females, (4) febrile within 2 weeks, (5) subjects with immune dysfunction, (6) subjects with organic psychosis, or (7) subjects diagnosed other psychiatric diagnoses such as disruptive mood dysregulation, persistent depressive disorder (dysthymia), premenstrual dysphoric disorder, bipolar depressive episode, etc.

Healthy volunteers were recruited as controls through matching demographic data on enrolled patients. These volunteers were (1) adults (20 to 65 years old) who did not meet any DSM-5 diagnostic criteria and (2) of normal intelligence. Subjects were excluded if they were: (1) unable to undergo MRI scanning, (2) left-handed, (3) pregnant females, (4) febrile within 2 weeks, (5) subjects with immune dysfunction, or (5) subjects with substance abuse or dependence history within one year, (6) subjects with a HAMD-17 score greater than or equal to 8 points, or (7) subjects with mental or genetic neurological disorders in first-degree relatives.

This study was approved by the ethics committee of the Guangji Hospital affiliated to Soochow University (ethics number SGLS2018-035). All participants provided written informed consent. All study procedures were in compliance with the Declaration of Helsinki.

### Blood sampling and ELISA test

Fasting peripheral blood was collected from the enrolled patients and controls. After centrifugation at 3000 rpm for 10 min, the supernatant was stored at -80 °C and the ELISA test was performed according to the kit manual to obtain the IL-18 concentration.

### Clinical assessment

The Chinese HAMD-17 Depression Rating Scale was used to assess depression due to its good reliability and validity [18]. Subjects with depressive disorders were defined as having a HAMD-17 score of no less than 8 points. Clinical assessments were completed by the same psychiatrist.

### MRI data acquisition

Imaging data were collected using Siemens 3.0 Tesla scanner (Skyra, Siemens, Germany) with a 32-channel head-coil. Before resting-state imaging, patients were scanned for anatomical positioning using T1 MPRAGE with parameters of 192 slices, 1 mm slice thickness, TR = 2530.0 ms, TE = 2.98 ms, FOV = 256 mm × 256 mm, and a voxel size of 1.0 mm × 1.0 mm × 1.0 mm. EPI sequence was used to acquire rs-MRI BOLD signal with parameters of 32 layers, 3.5 mm layer thickness, TR = 2000.0 ms, TE = 30.0 ms, FOV = 224 mm × 224 mm, matrix = 64 × 64, flip angle = 90°, and voxel size = 3.5 mm × 3.5 mm × 3.5 mm, 32 slices and 3.5 mm slice thickness, resulting in 240 volumes.

### Image data pre-processing

Data were pre-processed by the cortex-based resting-state fMRI data analysis package DPABISurf developed by DPABI/DPABISurf [19, 20]. DPABISurf calls fMRIprep to pre-process the structural and functional magnetic resonance data and provides a series of statistical and visualization tools through fMRIprep.

The pre-process is as follows. First of all, the first 10 time points were removed to ensure the uniformity of the magnetic field signal and the data was converted to BIDS format and call fmriprep1.5.0 docker.

Then, for preprocessing of structural image data, N4BiasFieldCorrection was used to correct the unevenness of the T1 weighted image, the corrected T1 weighted image was set as the reference T1 weighted image, OASIS30ANT was used as a template, the antBrainExtraction tool (from ANTs) was used to convert the T1 image into skull removal, the T1 image from the skull was segmented into cerebrospinal fluid (CSF), white matter (WM) and gray matter (GM) through the FAST method, and the recon-all command was taken to reconstruct the brain surface.

Next, the functional image data was preprocessed which contains generating a reference image and remove its skull through fMRIPrep’s custom method, registering the BOLD image with the structural image through the boundary-based registration method (from FreeSurfer), correcting the time level of the BOLD sequence by using 3dTshift, and setting the BOLD Resample the time series to the surface on fsaverage5 space.

Afterwards, Nuisance regression was performed by using the Friston-24 parametric model to remove the effects of head movement. In addition, in the group analysis, the average FD was used to regress out the residual effects of head motion. Other noise sources (white matter and cerebrospinal fluid signals) were also removed from the data by linear regression to reduce respiratory and cardiac effects. Besides, the linear trend was included in the regression to remove the influence of blood oxygen level-dependent scanner drift in the signal.

Finally, filtering and smoothing were performed by using 0.01–0.1 Hz band-pass filter and the images were smoothed with a kernel of 6 mm FWMH.

Pre-processed image results were processed through online quality control to eliminate subjects with large T1 structural image translations and fuzzy functional images with poor quality or incomplete functional image coverage. Subject data with head movements greater than 3 mm and 3 degrees were excluded and the remaining data were retained for statistical analysis.

In this study, we calculated the FC between DC and the vertex level of the whole cerebral cortex by vertex, and then took the positive connection of FC exceeding the threshold value (*r* > 0.25 in this study) to obtain the weighted sum of the whole cerebral DC map at the cortical level [13]. In terms of DC analysis, the value of DC was calculated using DPABISurf on the Matlab (R2018b) platform. For each vertex, we extracted the BOLD time series that depend on blood oxygen levels and calculate the Pearson’s correlation coefficient for vertices. Obtain the matrix of pearson correlation coefficients between any pair of vertices to construct Whole-brain functional connectivity matrix for each participant. The matrix is binarized with a threshold of *r* > 0.25. Finally, the resulting matrix (DC map) was smoothed using a Gaussian smoothing kernel for group comparison.

### Statistical analysis

The differences between groups of normally distributed continuous, non-normally distributed continuous, and categorical variables used two independent sample t-tests, the Mann–Whitney-rank sum test, and chi-square analysis, respectively. Using SPSS 24.0, the data were statistically analyzed with a two-tailed statistical significance set at *p* < 0.05.

The relationship between resting-state fMRI data and serum IL-18 levels was analyzed with DPABISurf statistical analysis using Matlab 2017b software. Pearson correlation was performed and corrected through threshold-free cluster enhancement (TFCE) to determine whether brain region differences between groups were present at the whole-brain level. The false positive rate (family wise error rate) level was controlled at *p* < 0.025 and the Bonferroni correction was performed on the two brain hemispheres.

## Results

**Fig. 1:**
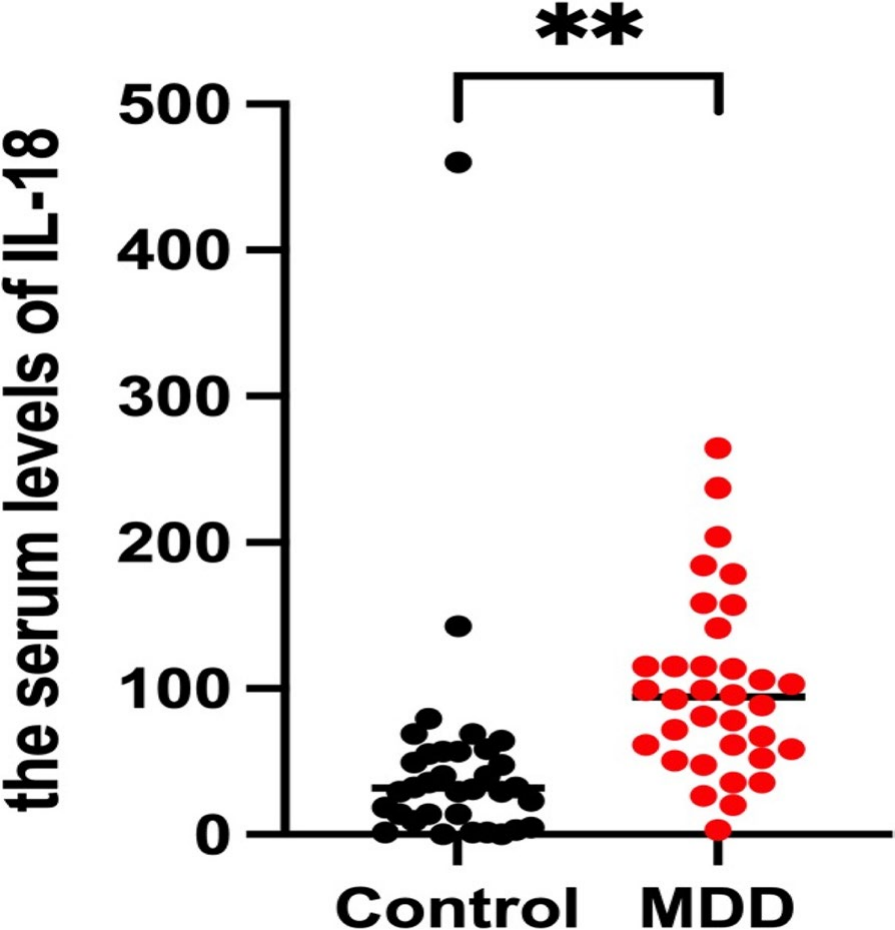
The serum levels of IL-18 in patients with depression and healthy people in the Peripheral blood. MDD: major depressive disorder, Control: healthy control; **: < 0.01

A total of 37 depression patients (10 men and 27 women) and 35 demographic-matched healthy controls (8 men and 27 women) were recruited for the study. The following subjects were excluded from the study: one male patient diagnosed with bipolar disorder during the study, one male and one female patient who was unable to undergo an MRI scan due to discomfort, and one healthy female with head movement exceeding 3 mm. Thus, a total of 68 patients (34 depression patients and 34 healthy controls) were included in the study (Table 1). As expected, the HAMD-17 score was also significantly higher in patients with depression (*P* < 0.001). Pearson correlation analysis showed a positive correlation between IL-18 and age in patients with depression (*r* = 0.403, *P* = 0.018). The serum levels of IL-18 in patients with depression and healthy people can be seen in the Fig. 1 and serum IL-18 levels were statistically increased in patients with depression compared to healthy controls (Z = -4.729, *P* < 0.001).

**Table 1:** General demographic data and clinical characteristics of the patient and the normal control

Variable	MDD	HC	χ^2^/Z	*P* value
Gender(M/F)	8/26	8/26	0.000	1.000
Age(years)	43.74 ± 14.09	41.09 ± 12.44	-0.890	0.373
Education(years)	10.41 ± 5.48	11.12 ± 4.42	-0.316	0.752
HAMD-17	18.41 ± 7.82	1.18 ± 1.83	-7.184	< 0.001
IL-18	100.54 ± 61.24	47.48 ± 78.76	-4.729	< 0.001

**Fig. 2:**
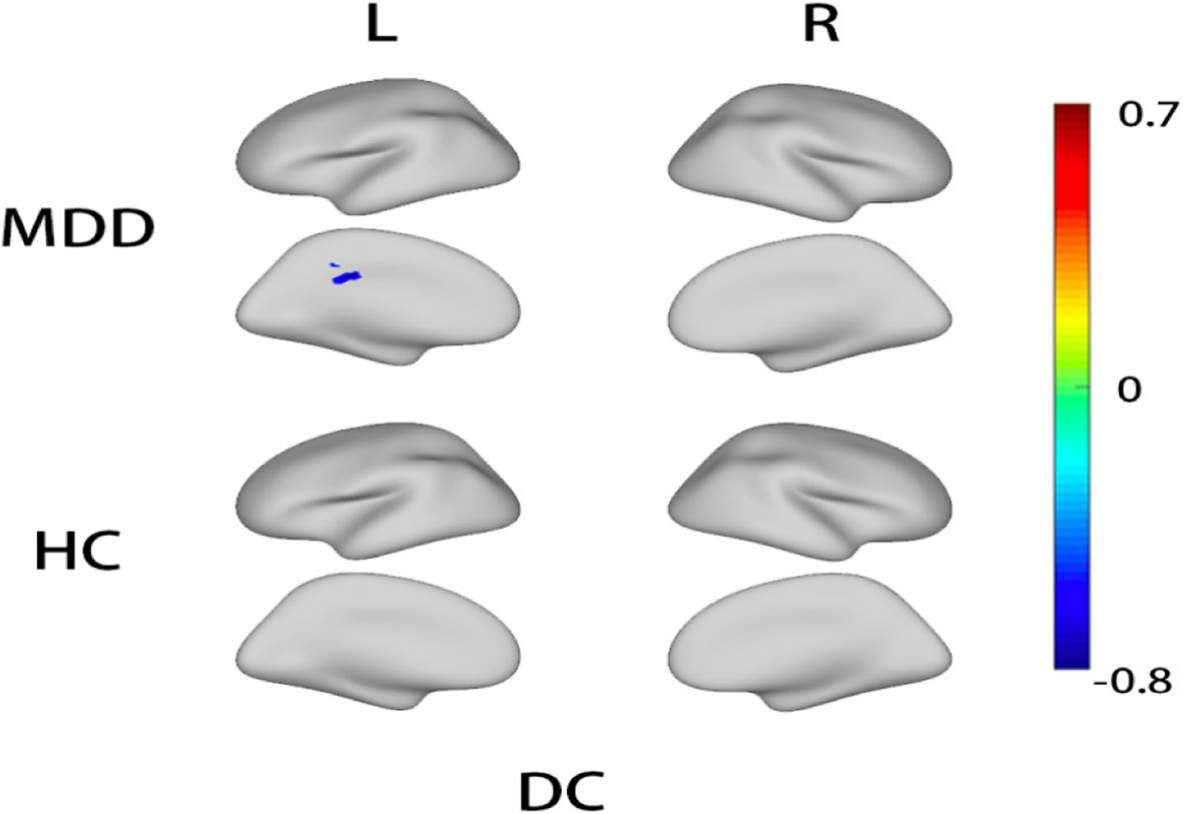
Correlation between IL-18 levels and DC in patients with depression and healthy people in the whole brain. Blue clusters are the brain area with negative correlation. MDD: major depressive disorder, HC: healthy control; L: left hemisphere; R: right hemisphere

**Fig. 3:**
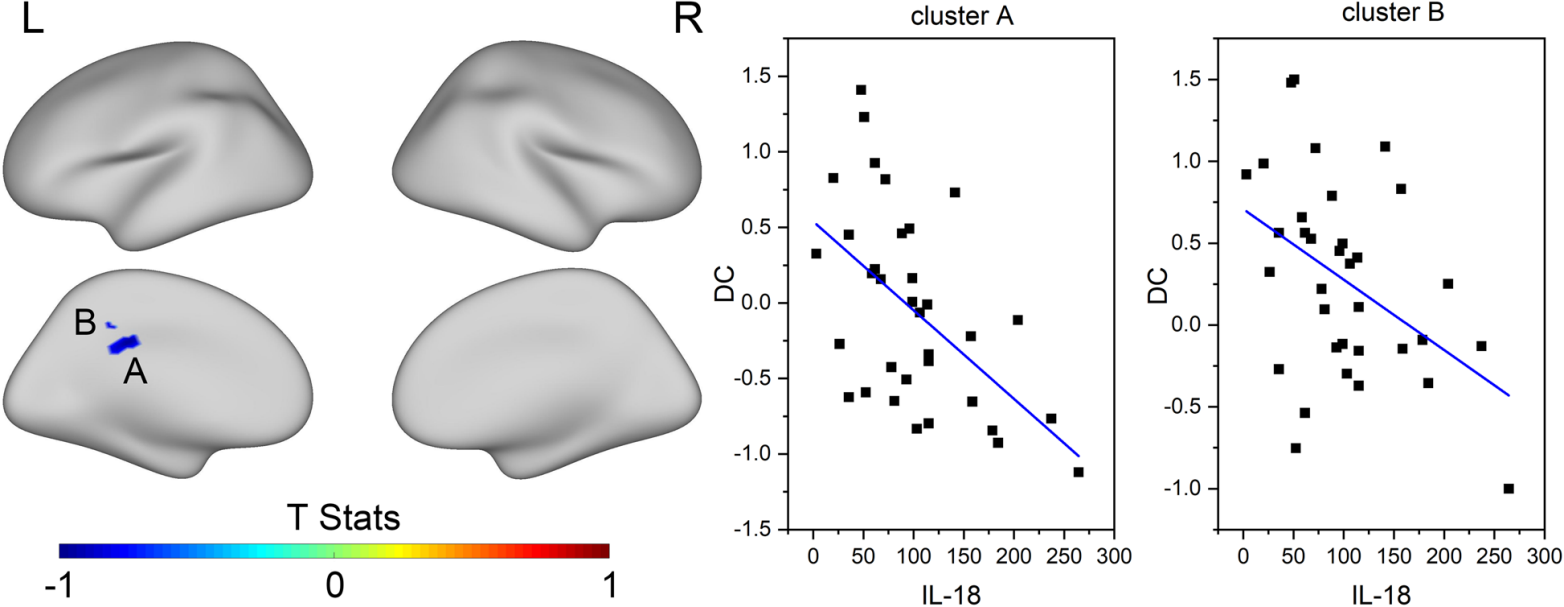
IL-18 and DC in brain imaging of patients with depression

Pearson correlation analysis of serum IL-18 level and resting-state fMRI imaging using covariates of patient age, gender, and years of education was completed to determine differences at the whole-brain level between depression patients and healthy controls. The correction was made by the replacement test processed by TFCE. The false-positive rate was controlled at *p* < 0.025 and Bonferroni correction was performed on both hemispheres of the brain. DC of the left posterior cingulate gyrus was negatively correlated with IL-18 in patients with depression (*p* < 0.05), and the specific correlated locations are the cluster A (34, -19,23) and cluster B (30, -34,33) marked in Fig. 3. However, there was no correlation between DC and the HAMD-17 score. Further results and specific correlations can be seen in Figs. 2 and 3.

## Discussion

To the best of our knowledge, this is the first study to investigate the correlation between the IL-18 and DC. Furthermore, we explored the possible mechanism of IL-18-mediated depression and interference with depression-related brain activity. In patients with depression, DC of the left posterior cingulate gyrus was significantly and negatively correlated with serum IL-18 levels. This association may be related to the neuronal activity of the left cingulate gyrus. Furthermore, the correlation was not found in healthy controls.

This study found that the level of IL-18 in patients with depression was significantly higher than that in healthy controls. As a pro-inflammatory cytokine of the IL-1 superfamily, IL-18 can synthesize several other cytokines including TNF-α and IL-1β [21]. Additionally, IL-18 plays a vital role in the regulation of innate and adaptive immunity, where abnormal regulation may cause various autoimmune diseases [22, 23]. As an important regulator of immune activity, IL-18 has been found to be related to the occurrence of schizophrenia [24], and may also be involved in the pathogenesis of depression.

Peripheral inflammation is highly associated with depressive symptoms, increasing the risk for major depressive disorder. However, the exact mechanism of inflammation-induced depression remains unclear.

The posterior cingulate gyrus is highly connected and metabolically active [25] component of the default network [26]. As the default network is closely related to cognitive function, the posterior cingulate gyrus is involved with attention, executive, and cognitive function [27–30]. It is hypothesized that the posterior cingulate gyrus plays a pivotal role in supporting internally directed cognition [26, 31]. Several studies have shown periods of “cognitive freedom” with increase cingulate gyrus cortex activity during periods of autobiographical memory recall and non-controlled brain activity [32–34]. However, other studies suggest that the posterior cingulate gyrus plays a more direct role in adjusting attention focus – internal idea retrieval, episodic and semantic memory, and behavioral responses to external environments [35, 36]. The posterior cingulate gyrus has been proposed to regulate the metastable state of the whole brain internal network, adjust brain activity stability, and control the degree of change in neural activities – all of which affect attention [37]. Additionally, changes in posterior cingulate cortex (PCC) activity with arousal and its interaction with other neural networks may suggest an important role in conscious awareness [38].

The results of this study showed that IL-18 and DC values were significantly negatively correlated in the left posterior cingulate gyrus. Degree centrality, an important resting state brain imaging indicator, is closely related to whole-brain functional connectivity and can reflect the importance of specific brain node or area relative to the whole brain network [12, 13]. Prior imaging studies have suggested that patients with depression have abnormalities in the structure and function of the posterior cingulate gyrus. One study highlighted the reduction in functional connectivity from the posterior cingulate back to the caudate body in patients with depression [39]. In depression, enhanced PCC and sgACC activity are associated with excessive ruminant thinking, a behavioral characteristic of the disorder [40]. Several studies have shown that ruminative thinking is a risk factor for the development and maintenance of depression [41–43].

One widely accepted hypothesis is the interference of inflammation on neurotransmitters (serotonin, dopamine, and norepinephrine) within brain neural circuits that induce emotional responses. This is suggested to be due to the activity of pro-inflammatory cytokines (including IL-18) on indoleamine-2,3-dioxygenase in reducing extracellular serotonin (5-HT) [44]. Studies show that 5-HT subtypes (5-HT1A and 5-HT2A) both affect the default mode network (DMN) – suggesting its close relationship with clinical manifestations of depression [45, 46]. While the DMN is known to be closely related to rumination and depression, the core component involves the posterior cingulate gyrus [26]. Thus, serotonin may also affect the posterior cingulate gyrus. Furthermore, recent studies have demonstrated an interaction between IL-18 and 5-HT may cause alexithymia [47]. Thus, IL-18, a peripheral pro-inflammatory cytokine, may affected individual brain neurons through 5-HT modulation inducing corresponding changes in brain activity and the manifestation of affective disorders.

The current study found that IL-18 showed a significant and negative correlation with DC values in the left posterior cingulate gyrus. This suggests that elevated IL-18 may affect 5-HT to cause neural circuit modulation in the left cingulate gyrus and interference in the back left cingulate region. Furthermore, abnormal levels of IL-18 may also be related to the pathogenesis of depression. The DC level in the left posterior cingulate gyrus showed a significant and negative correlation with IL-18 in depression patients. This suggests that abnormal levels of IL-18 in the peripheral blood may interfere with functional brain activity – suggesting a relationship between depression onset and IL-18. As the brain is highly connected with surrounding regions, these changes suggest a decrease in signal and hub function in the primary affected neural region and its surrounding connected brain areas. These brain activity changes may be related to an individual’s attention, executive, memory, and emotional function. These decreases in regulatory functions may be related to depression-like manifestations – suggesting that IL-18 mediates the pathogenesis of depression through an inflammatory response. In other words, the findings in this study suggest IL-18 may be one of the causes of spontaneous brain activity abnormalities in the posterior cingulate gyrus, and it may be due to spontaneous brain activity abnormalities in the posterior cingulate gyrus, which leads to the impairment of neural circuit functions such as emotion involved, and causes depression. However, in this study, we did not find that DC and IL-18 were associated with HAMD-17 levels. This result is difficult to interpret, which may be due to the fact that the small sample size and sample quality had a greater impact on the results of this study.

## Limitations and conclusion

The limitations of this study were as follows. First of all, this study is only a cross-sectional study, and no follow-up study was performed on the patients. It cannot explain whether the relationship between the level of IL-18 and DC in patients with depression is the cause of the disease or the consequence of the disease. Future research could further explore the relationship between the clinical symptom evolution of patients with depression before and after intervention and the changes of immune inflammation and brain function. Second, the sample size of the study is small, and the representativeness of the results is limited. Future research needs to further expand the sample size and repeat the experiment to verify the results. At last, depression is only one of the common inflammation-related mental diseases. It is not clear whether these research results can be extended to other mental diseases, such as schizophrenia and bipolar disorder.

In conclusion, the negative correlation between IL-18 and DC in the left cingulate gyrus suggests that IL-18 causes regional interference and dysfunction, resulting in depression-like manifestations such as low mood and reduced executive function. This may suggest that IL-18 mediates the pathophysiological mechanism of depression and interferes with brain activity.

## Data Availability

The datasets generated and analysed during the current study are not publicly available due to protect the subjects’ privacy and to fully analyze the data in the future, but they are available from the corresponding author Yansong Liu on reasonable request. E-mail address: lysway@163.com.

## Abbreviations

DC: Degree centrality
MDD: Major depressive disorder
IL-18: Interleukin-18
IL-6: Interleukin-6
MRI: Magnetic Resonance Imaging
fMRI: Functional Magnetic Resonance Imaging (fMRI)
HAMD-17: Hamilton depression-17 (HAMD-17)
ELISA: Enzyme-linked immunosorbent assay
